# Postoperative radiotherapy for pathological stage IIIA‐N2 non‐small cell lung cancer with positive surgical margins

**DOI:** 10.1111/1759-7714.13749

**Published:** 2020-11-27

**Authors:** Meng Yuan, Yu Men, Jingjing Kang, Xin Sun, Maoyuan Zhao, Yongxing Bao, Xu Yang, Shuang Sun, Zeliang Ma, Jianyang Wang, Lei Deng, Wenqing Wang, Yirui Zhai, Wenyang Liu, Tao Zhang, Xin Wang, Nan Bi, Jima Lv, Jun Liang, Qinfu Feng, Dongfu Chen, Zefen Xiao, Zongmei Zhou, Luhua Wang, Zhouguang Hui

**Affiliations:** ^1^ Department of Radiation Oncology; ^2^ Department of VIP Medical Services, National Cancer Center/National Clinical Research Center for Cancer/Cancer Hospital, Chinese Academy of Medical Sciences and Peking Union Medical College Beijing China

**Keywords:** NSCLC, pIIIA‐N2, positive surgical margins, postoperative radiotherapy

## Abstract

**Background:**

The aim of this study was to evaluate the efficacy of postoperative radiotherapy (PORT) in stage pIIIA‐N2 non‐small cell lung cancer (NSCLC) patients with positive surgical margins.

**Methods:**

Between January 2003 and December 2015, patients who had undergone lobectomy or pneumonectomy plus mediastinal lymph node dissection or systematic sampling in our single institution were retrospectively reviewed. Those with pIIIA‐N2 NSCLC and positive surgical margins were enrolled into the study. The Kaplan‐Meier method was used for survival analysis, and the log‐rank test was used to analyze differences between the groups. Univariate and multivariate analyses using Cox proportional hazards regression models were performed to evaluate potential prognostic factors for OS. Statistically significant difference was set as *P* < 0.05.

**Results:**

Of all the 1547 patients with pIIIA‐N2 NSCLC reviewed, 113 patients had positive surgical margins, including 76 patients with R1 resection and 37 with R2 resection. The median overall survival (OS) was 28.3 months in the PORT group and 22.6 months in the non‐PORT group (*P* = 0.568). Subset analysis showed that for patients with R1 resection, the median OS was 52.4 months in the PORT group which was nonsignificantly longer than that of 22.6 months in the non‐PORT group (*P* = 0.127), whereas PORT combined with chemotherapy could significantly improve OS, with a median OS of 52.4 months versus 17.2 months (*P* = 0.027). For patients with R2 resection, PORT made no significant difference in OS (17.6 vs. 63.8 months, *P* = 0.529).

**Conclusions:**

For pIIIA‐N2 NSCLC patients with positive surgical margins, PORT did not improve OS, but OS was improved in those patients who underwent R1 resection combined with chemotherapy.

**Key points:**

**Significant findings of the study:**

Significant findings of the study: Postoperative radiotherapy (PORT) has been recommended to treat patients with positive surgical margins. However, the existing evidence is controversial and high‐level evidence is lacking.

**What this study adds:**

What this study adds: The PORT group had markedly, but not statistically significant, longer median OS compared with the non‐PORT group in patients with R1 resection. OS was significantly longer in the patients with R1 resection receiving adjuvant CRT than the surgery alone group.

## Graphical abstract

Postoperative radiotherapy (PORT) has been recommended to treat patients with positive surgical margins. However, the existing evidence is controversial and high‐level evidence is lacking. In our study, the PORT group had markedly, but not statistically significant, longer median OS compared with the non‐PORT group in R1 resection patients. OS was significantly longer in the R1 resection patients receiving adjuvant CRT than the surgery alone group.

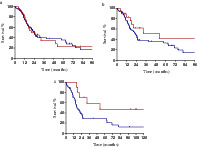

## Introduction

Surgery is the mainstay of curative treatment for operable non‐small cell lung cancer (NSCLC). Although intraoperative frozen section evaluation has been routinely performed, some patients are left with microscopic (R1 resection) or macroscopic (R2 resection) residual tumor at the surgical margins, especially among those with pN2,^1^, ^2^, ^3^ with an incidence rate of 1%–17%.^4^, ^5^ An incomplete resection can significantly compromise locoregional control, as well as long time overall survival (OS).^2^, ^4^, ^5^ Postoperative radiotherapy (PORT) has been recommended to treat patients with positive surgical margins, while high‐level clinical evidence is lacking. Some historical studies showed that PORT was associated with improved OS,^1^, ^6^ whereas others did not support the use of PORT,^2^, ^7^, ^8^ which may due to outdated two‐dimensional radiotherapy techniques with high toxicities, or the improper selection of low risk patients. Therefore, the aim of this study was to evaluate the efficacy and safety of PORT on the survival of pIIIA‐N2 NSCLC patients with positive surgical margins using modern radiotherapy techniques.

## Methods

### Patient selection and data collection

In this retrospective case control study, inclusion criteria were as follows: (i) Patients undergoing surgery in our center between January 2003 and December 2015; (ii) with postoperative microscopic or macroscopic residual disease identified; (iii) pathologically diagnosed as stage IIIA‐N2 NSCLC, according to the seventh edition of the American Joint Committee on Cancer Staging System; and (iv) complete medical records available. Study variables included gender, age, smoking history, tumor location, surgery type, status of surgical margins, histological subtype, tumor size, number of positive lymph nodes and postoperative treatment.

### Postoperative radiotherapy (PORT)

Radiotherapy techniques included intensity modulated radiotherapy (IMRT) and three‐dimensional conformal radiotherapy (3DCRT) using 6 MV X‐rays. All patients underwent computed tomography (CT) simulation for PORT. The gross tumor volume (GTV) was defined as the area of residual tumor (those that were well located). The clinical target volume (CTV) enclosed the GTV with a 6 or 8 mm margin, bronchial stump, subcarinal nodes, ipsilateral mediastinum and ipsilateral hilum. The planning target volume (PTV) comprised the CTV with a 5 mm margin. The main dose‐volume constraints (DVCs) for lung were set as follows: V20 <20% for lobectomy or <10% for pneumonectomy. The prescription dose of PTV was 60–66 Gy, with a conventional‐fraction schedule.

### Adjuvant chemotherapy

Platinum‐based double‐agent regimens of 4–6 cycles were recommended for patients, and some were given concurrently or sequentially with PORT.

### Adverse events

We used the National Cancer Institute Common Toxicity Criteria for Adverse Events version 4.0 (CTCAE 4.0) to assess side effects.

### Statistical analysis

OS was calculated from surgical resection until death or last follow‐up. Progression‐free survival (PFS) was measured from surgical resection to failure at any site, death or last follow‐up. Local progression‐free survival (LPFS) was calculated from surgery to recurrence at the surgical resection margins, death or last follow‐up. Regional recurrence‐free survival (RRFS) was defined as the duration from surgery to mediastinal relapse (except the surgical margins), death or last follow‐up. Distant metastasis‐free survival (DMFS) was defined as the time between surgery and recurrence in any other organ, contralateral lobe or malignant pleural/pericardial effusion, death or last follow‐up. SPSS version 25.0 (IBM) was used to perform the statistical analysis. Kaplan‐Meier method was used for survival analysis, and log‐rank test was adopted to analyze differences. Cox proportional hazards regression models were applied in univariate and multivariate analyses to evaluate potential prognostic factors for OS. A *P*‐value of less than 0.05 was set as the threshold for significance.

## Results

### Patient demographic and clinicopathological characteristics

Of all the 1547 patients with pIIIA‐N2 NSCLC receiving lobectomy or pneumonectomy plus mediastinal lymph node dissection or systematic sampling, 113 (7.3%) were diagnosed with positive surgical margins, including 76 (67.3%) with R1 resection and 37 (32.7%) with R2 resection. As for the sites of microscopic residual tumors for R1 resection, 70 cases (92.1%) were at the bronchial stumps, three at lymph nodes and three at the vessel walls. For R2 resection, the sites of macroscopic residual tumors were as follows: 22 cases (59.5%) at the bulky metastatic lymph nodes, 11 (29.7%) at the bronchial stumps, four (10.8%) at the vessel walls and one at the pericardium. The median age was 58 (range: 38–83) years. Adenocarcinoma was identified in 57 patients (50.4%), followed by squamous cell carcinoma in 42 (37.2%), adenosquamous carcinoma in seven, large cell carcinoma in four and others in three. Of the 113 patients in this study, 36 (31.9%) received PORT. The demographic and clinicopathological details between the PORT and non‐PORT groups are presented in Table 1. The factors were comparable between the PORT and non‐PORT groups, except that there were more patients receiving chemotherapy (66.7% vs. 28.6%), and less R1 resection (50.0% vs. 75.3%) or non‐squamous cell carcinoma patients (50.0% vs. 68.8%) in the PORT group.

**Table 1:** Patient demographic and clinicopathological characteristics

	Total		PORT		Non‐PORT			
Factors	*n*	%	*n*	%	*n*	%	χ^2^	*P*‐value
Age							0.70	0.40
≤60 years	61	54.0	22	61.1	39	50.6		
>60 years	52	46.0	14	38.9	38	49.4		
Gender							0.00	1.00
Male	91	80.5	29	80.6	62	80.5		
Female	22	19.5	7	19.4	15	19.5		
Smoking history							0.00	1.00
Yes	78	69.0	25	69.4	53	68.8		
No	35	31.0	11	30.6	24	31.2		
Tumor location							3.87	0.49
Left lung	38	33.6	7	19.4	31	40.3		
Right lung	75	66.4	29	80.6	46	59.7		
Type of surgery							0.00	1.00
Lobectomy	89	78.8	28	77.8	61	79.2		
Pneumonectomy	24	21.2	8	22.2	16	20.8		
Surgical margins							6.04	0.01
R1 resection	76	67.3	18	50.0	58	75.3		
R2 resection	37	32.7	18	50.0	19	24.7		
Histology subtype							13.21	0.01
SCC	42	37.2	18	50.0	24	31.2		
Non‐ SCC	71	62.8	18	50.0	53	68.8		
Pathological T stage							5.81	0.12
T1/T2	73	64.6	19	52.8	54	70.1		
T3/T4	40	35.4	17	47.2	23	29.9		
Positive lymph nodes							2.59	0.28
≤4	42	37.2	17	47.2	25	32.4		
4–10	41	36.3	12	33.3	29	37.7		
>10	30	26.5	7	19.5	23	29.9		
Adjuvant chemotherapy							14.75	0.00
Yes	46	40.7	24	66.7	22	28.6		
No	67	59.3	12	33.3	55	71.4		

SCC, squamous cell carcinoma.

### Treatment

Of all patients included in this study, 89 (78.8%) patients underwent lobectomy and 24 (21.2%) underwent pneumonectomy. For 36 patients receiving PORT, the median total dose of PORT was 60 (range: 45–70) Gy including two patients who failed to complete the prescription dose due to distant metastasis (the doses received were 45 and 50 Gy, respectively). A total of 46 (40.7%) patients received chemotherapy with a median course of four (range: 1–6). In this study, 24 (21.2%) patients received chemoradiotherapy (CRT), including seven with concurrent CRT, 15 with chemotherapy followed by radiotherapy and two with radiotherapy followed by chemotherapy.

### Survival impact of PORT on patient outcomes

**Figure 1:**
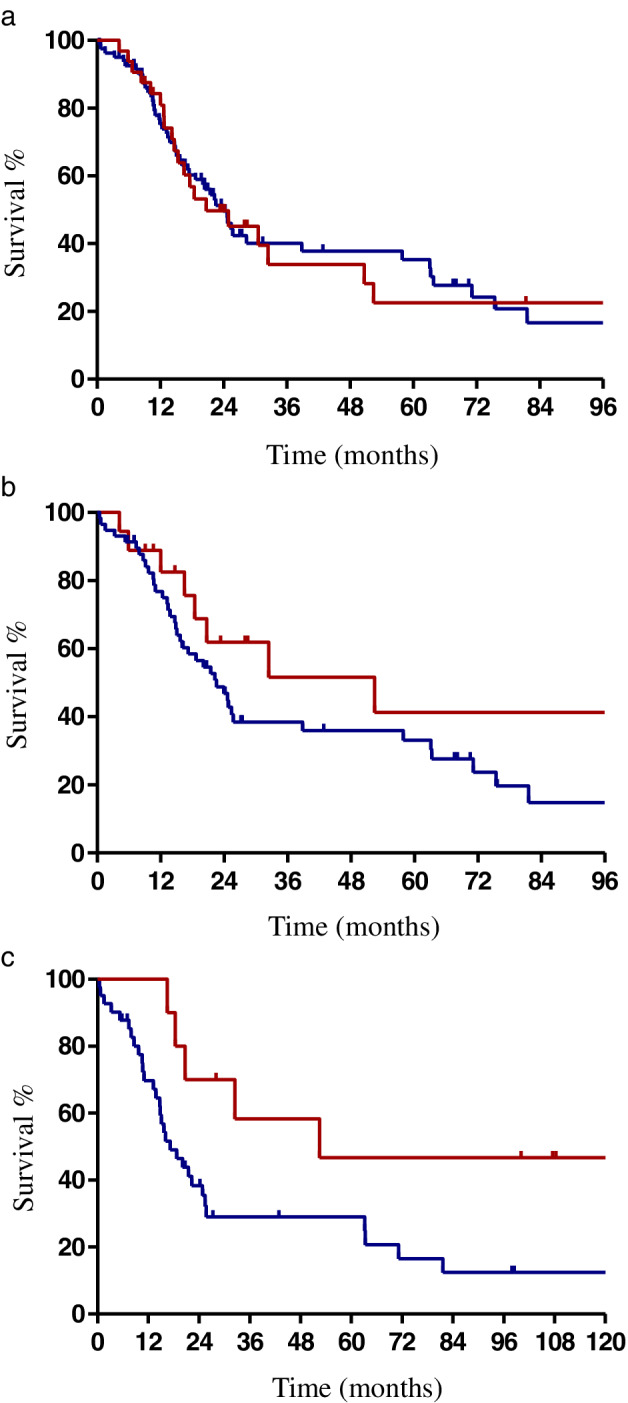
Effect of PORT or adjuvant CRT on overall survival. (**a**) All patients. (**b**) Patients with R1 resection. (**c**) Patients with R1 resection. (**a**) (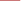 ) PORT, (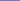 ) non‐PORT; (**b**) (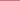 ) PORT, (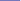 ) non‐PORT; (**c**) (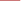 ) Adjuvant CRT, (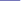 ) Surgery alone.

The median follow‐up was 19.8 (range: 0.5–165.8) months. A total of 70 (61.9%) patients died during follow‐up. The median OS was 23.9 months and the median PFS was 17.1 months of all patients. Survival analysis showed that the median OS in PORT group (28.3 months) was a little longer than non‐PORT group (22.6 months) with no significant difference (*P* = 0.568, Fig 1a). No significant differences of LRFS (24.9 vs. 20.6 months, *P* = 0.466), RRFS (25.3 vs. 21.8 months, *P* = 0.655) or DMFS (12.1 vs. 14.8 months, *P* = 0.710) were demonstrated between the PORT and non‐PORT groups.

Univariate analysis was performed according to patient clinicopathological characteristics. The detailed results are shown in Table 2. Younger patients (age 60 years or less) (HR = 0.561, *P* = 0.018) and receiving chemotherapy (HR = 0.594, *P* = 0.037) were significantly associated with better OS. In the multivariate analysis, only age was the independent prognostic factors for OS (HR = 0.561, *P* = 0.047) (Table 2).

**Table 2:** Prognostic factors by univariate and multivariate analyses

	Univariate analysis			Multivariate analysis		
Factors	HR	95% CI	*P*‐value	HR	95% CI	*P*‐value
Age			0.018^*^			0.047^*^
≤60 years	0.561	0.348–0.958		0.561	0.317–0.993	
>60 years						
Gender			0.483			0.152
Male	1.260	0.660–2.407		1.830	0.801–4.177	
Female						
Smoking history			0.297			0.688
Yes	1.319	0.784–2.217		1.148	0.586–2.249	
No						
Tumor location			0.972			0.744
Left lung	1.009	0.615–1.655		0.912	0.527–1.581	
Right lung						
Type of surgery			0.769			0.278
Lobectomy	0.920	0.525–1.610		0.681	0.340–1.364	
Pneumonectomy						
Surgical margins			0.374			0.626
R1 resection	0.792	0.473–1.325		0.866	0.485–1.547	
R2 resection						
Histological subtype			0.217			0.110
SCC	1.368	0.832–2.250		1.695	0.888–3.237	
Non‐ SCC						
Pathological T stage			0.888			0.271
T1/T2	0.965	0.590–1.579		0.701	0.373–1.319	
T3/T4						
Positive lymph nodes			0.052			0.075
≤4	0.575	0.328–1.010		0.649	0.335–1.259	
4–10	0.502	0.275–0.915		0.480	0.254–0.905	
>10						
PORT			0.569			0.874
Yes	0.862	0.516–1.438		0.874	0.453–1.685	
No						
Adjuvant chemotherapy			0.037^*^			0.152
Yes	0.594	0.365–0.968		0.638	0.346–1.179	
No						

^*^ *P*‐value <0.05. PORT, postoperative radiotherapy.

When OS was assessed by surgical margin status, the PORT group had markedly, but not statistically significant, longer median OS of 52.4 months compared with the non‐PORT group of 22.6 months (*P* = 0.127, Fig 1b) in patients with R1 resection. However, in patients with R2 resection, the OS of the PORT group was not improved when compared with the non‐PORT group. When stratified by treatment patterns, the median OS was significantly longer in patients with R1 resection receiving adjuvant CRT (52.4 months) than surgery alone group (17.2 months) (*P* = 0.027, Fig 1c). OS was similar between adjuvant CRT and surgery alone groups in patients with R2 resection (24.9 vs. 20.4 months, *P* = 0.692). In patients receiving CRT, no significant difference was detected in OS between concurrent and sequential CRT (32.3 vs. 28.3 months, *P* = 0.890).

### Recurrence patterns

**Figure 2:**
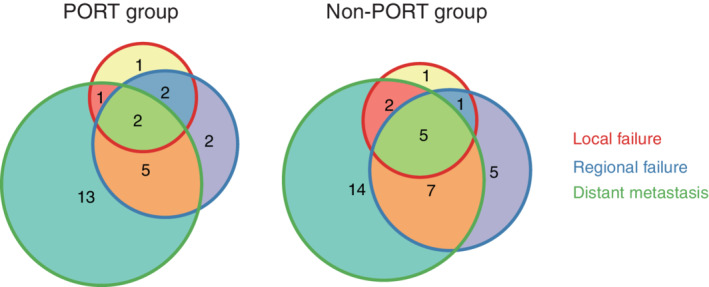
Recurrence patterns of PORT and non‐PORT groups.

In both the PORT and non‐PORT groups, distant metastasis was the most predominant type of relapse, accounting for 58.3% (*n* = 21) and 36.4% (*n* = 28), respectively. Six (16.7%) and nine (11.7%) patients in the PORT and non‐PORT groups were diagnosed with local progression. Eleven (30.6%) and 18 (23.4%) patients in the two groups suffered from regional recurrence. Compared with the non‐PORT group, PORT did not change the failure pattern in patients with positive surgical margins (Fig 2).

### Safety profile of PORT

As for the side effects, there were no grade 3 or higher adverse events in this study. A total of 10 (27.8%) cases of grade 1/2 esophagitis were reported in the PORT group, but for patients receiving concurrent CRT, the rate was relatively higher. Five of seven patients were diagnosed with esophagitis. There was no radiation pneumonitis observed.

## Discussion

In this study, although PORT did not improve the overall survival of pIIIA‐N2 NSCLC patients with positive surgical margins, the results still deserve further consideration and also provide reference for clinical practice.

According to the newly published NCCN guidelines,^9^ for IIIA‐N2 NSCLC patients with positive surgical margins, the recommended treatment strategies include sequential or concurrent CRT for R1 resection and concurrent CRT for R2 resection. However, the existing evidence is controversial and high‐level evidence is lacking.^1^, ^2^, ^6^, ^7^, ^8^ Moreover, among those early studies, many used two‐dimensional radiotherapy techniques or may have included the inappropriate selection of early‐stage patients.^7^, ^8^, ^10^, ^11^, ^12^, ^13^ In China, considering the potential serious adverse events and low completion rate, only a few patients with positive surgical margins received PORT, and even fewer received concurrent CRT. Our study showed that for all 113 patients, only 36 received PORT, including 17 with sequential CRT and only seven with concurrent CRT. This fact, however, presented an opportunity for conducting this retrospective case control study. In our study, only stage pIIIA‐N2 NSCLC patients were enrolled which was considered a high‐risk subset that could potentially benefit from adjuvant therapy,^1^, ^3^, ^14^ and PORT was given with the modern techniques of IMRT or 3DCRT. Nevertheless, the results indicated that PORT could not improve survival, and did not support the routine use of PORT. Our study also showed that there was no significant difference of OS between sequential and concurrent CRT groups, which was in accordance with the reports by Francis *et al*.^15^ and Smeltzer *et al*.^16^ However, due to the limited number of patients with CRT in our study, bias and uncertainty may exist.

For patients with R1 resection, the tumor burden is relatively low and the prognosis is favorable compared with R2 resection; therefore, local treatment should be administered more actively to enhance tumor control, coupled with systematic treatment to prolong survival. Our previously published study which included stage I–III NSCLC patients with R1 resection showed that S + R + C (PORT plus postoperative chemotherapy) significantly improved OS, compared with S (surgery) alone (median OS 47 vs. 16 months, *P* = 0.016), while not in S + R (PORT).^17^ Stage IIIA‐N2 NSCLC patients with R1 or R2 resection were included in the present study, and although PORT did not improve OS in the overall patient population, subgroup analysis showed that for patients with R1 resection, median OS was obviously, but not significantly, longer in the PORT than the non‐PORT group (52.4 vs. 22.6 months), which might be due to the relatively limited number of cases. When compared with surgery alone, adjuvant CRT significantly improved OS (median OS 52.4 vs. 17.2 months, *P* = 0.027). The result is in accordance with our previous study^17^ which emphasized that only when combined with chemotherapy was PORT able to effectively improve survival of patients who had undergone R1 resection.

With the intraoperative frozen section evaluation^18^ routinely performed in recent years, the incidence of R1 resection may decrease, and consequently, the ratio of R2 resection might be higher than ever, as shown in our study (ratio of R1 and R2 resection: 67.3% vs. 32.7%). However, many of the previous studies were only aimed at R1 resection^1^, ^19^, ^20^ or enrolled R1 resection cases.^6^, ^21^ We believe that to evaluate the effect of PORT for patients with R2 resection is necessary. In our study of 37 patients with R2 resection, 18 patients received PORT, among whom 14 received CRT but only four received concurrent CRT. The OS was similar with or without PORT. This may be due to the fact that there is a much higher tumor burden in patients with R2 resection, resulting in an even worse prognosis. Actually, in this study, most of the patients with R2 resection were those clinically diagnosed with stage IIIA‐N2 (78.4%). As for the reason for R2 resection, 59.5% (*n* = 22) cases were due to bulky metastatic lymph nodes which could not be completely resected. Additionally, postoperative immunosuppression may further contribute to the progression of residual disease.^22^, ^23^ For patients with R2 resection in our study, even postoperative radiotherapy could not make up for compromised patient survival following incomplete resection. Therefore, for most of the patients with clinical stage N2, especially those with bulky metastatic lymph nodes, the conclusion is that optimal treatment is concurrent chemoradiotherapy rather than surgery.

Our analysis showed that distant metastasis was the most predominant type of relapse, accounting for 58.3% in the PORT group and 36.4% in the non‐PORT group. This reflects the nature of stage III disease, which is more aggressive and prone to distant metastasis, and thus, adjuvant chemotherapy could, in part, compromise the risk of failure. Previous retrospective studies also reported that for patients with positive surgical margins, adjuvant chemotherapy could significantly improve OS.^3^, ^6^, ^17^, ^24^ Our study showed that chemotherapy was significantly associated with better OS in the univariate (HR = 0.594, *P* = 0.037), but not in the multivariate analysis. This in combination with the recurrence pattern mainly seen in patients with distant metastasis indicates that adjuvant chemotherapy should be strengthened or more precise treatment such as adjuvant TKIs for those with *EGFR* mutations is needed.^25^, ^26^

As for the safety profile of PORT, a total of 27.8% patients with grade 1/2 radiation esophagitis were reported and no radiation pneumonitis was observed in our study. The low incidence of PORT‐related adverse events could be due to the strict dose‐volume constraints with IMRT or 3DCRT techniques and one third of patients received radiotherapy without chemotherapy. In patients receiving concurrent CRT, there was a relatively high incidence rate of esophagitis (71.4%), but all of the cases were grade 1/2.

As a retrospective analysis, this study had some limitations. First, all patients came from a single institution and the sample size was limited, although to our knowledge, this is the only study aimed at patients with stage pIIIA‐N2 NSCLC with positive margins. Second, the ratio of patients receiving concurrent CRT was low, which was different from the recommendation of the guidelines, by reason of the low patient acceptance and tolerance in Chinese clinical practice. In addition, with the development of immunotherapy and targeted therapy in cancer treatment, the role of local radiotherapy needs to be re‐evaluated.

In conclusion, for pIIIA‐N2 NSCLC patients with positive surgical margins in this study, PORT did not improve OS, although was found to improve OS of those with R1 resection when combined with chemotherapy. The result is insufficient to support the recommendation of routine use of PORT. Further prospective studies are warranted to identify the value of PORT for NSCLC patients with positive surgical margins.

## Disclosure

The authors declare that they have no known competing financial interests or personal relationships.
